# Effects of Wharton’s Jelly Mesenchymal Stem Cells and Its-Derived Small Extracellular Vesicles Loaded into Injectable Genipin-Crosslinked Gelatin Hydrogel on Vocal Fold Fibroblast

**DOI:** 10.3390/polym17192653

**Published:** 2025-09-30

**Authors:** Zarqa Iffah Zamlus, Mawaddah Azman, Yogeswaran Lokanathan, Mh Busra Fauzi, Marina Mat Baki

**Affiliations:** 1 Department of Otorhinolaryngology-Head and Neck Surgery, Faculty of Medicine, Universiti Kebangsaan Malaysia, Kuala Lumpur 56000, Malaysia; zarqaifh98@gmail.com (Z.I.Z.); mawaddah@ukm.edu.my (M.A.); 2 Department of Tissue Engineering and Regenerative Medicine, Faculty of Medicine, Universiti Kebangsaan Malaysia, Kuala Lumpur 56000, Malaysia; lyoges@ppukm.ukm.edu.my (Y.L.); fauzibusra@ukm.edu.my (M.B.F.); 3 Advance Bioactive Materials-Cells UKM Research Group, Universiti Kebangsaan Malaysia, Bangi 43600, Malaysia

**Keywords:** small extracellular vesicles, Wharton’s Jelly mesenchymal stem cells, gelatin hydrogel, vocal fold fibroblasts, glottic insufficiency, vocal fold palsy

## Abstract

Glottic insufficiency, often caused by laryngeal nerve injury, impairs voice quality and breathing. Current treatments, such as hyaluronic acid injection, require frequent reapplication every 3–6 months. This study aimed to investigate the therapeutic potential of small extracellular vesicles (sEVs) derived from Wharton’s Jelly mesenchymal stem cells (WJMSCs) incorporated into genipin-crosslinked gelatin hydrogels (GCGHs) for promoting vocal fold fibroblast (VFFs) regeneration in vitro. WJMSCs were isolated from umbilical cords, expanded to passage 4, and used for sEV isolation via tangential flow filtration (TFF). The sEVs (585.89 ± 298.93 µg/mL) were characterized using bicinchoninic acid assay (BCA), nanoparticle tracking analysis (NTA), transmission electron microscopy (TEM), and Western blot. Seven concentrations of sEVs were tested on VFFs to evaluate cytotoxicity and proliferation, identifying 75 µg/mL as the optimal dose. GCGHs were then combined with WJMSCs and sEVs and evaluated for physicochemical properties, degradation, biocompatibility, and immune response. The hydrogels were injectable within 20 min and degraded in approximately 42 ± 0.72 days. The optimal sEV concentration significantly enhanced VFFs proliferation (166.59% ± 28.11) and cell viability (86.16% ± 8.55, *p* < 0.05). GCGH-MSCs showed the highest VFFs viability (82.04% ± 10.51) and matrix contraction (85.98% ± 1.25) compared to other groups. All hydrogel variants demonstrated minimal immune response when co-cultured with peripheral blood mononuclear cells (PBMCs). GCGH is a promising scaffold for delivering WJMSCs and sEVs to support VFF regeneration, with demonstrated biocompatibility and regenerative potential. Further in vivo studies are warranted to validate these findings.

## 1. Introduction

Voice production is essential for communication, emotional expression, and social interaction [1]. However, glottic insufficiency, a condition characterized by incomplete closure of the vocal folds, can severely impair these functions, leading to breathy voice, vocal fatigue, aspiration risks, and reduced phonatory efficiency [2,3]. This condition is commonly observed in patients with unilateral vocal fold paralysis or paresis, which can result from surgical trauma, ageing, neurological disorders, or systemic conditions affecting nerve or muscle function. Approximately 16.9% of persons aged 18 and over suffer from voice abnormalities, with incidence rates rising to 29.1% in those over 60, and this prevalence is noticeably high across all age categories [4,5,6].

Current treatment strategies for glottic insufficiency primarily include voice therapy, injection laryngoplasty, and medialization thyroplasty. Injection laryngoplasty, where biomaterials such as hyaluronic acid (HA), autologous fat, and calcium hydroxyapatite (CaHA) are injected into the vocal fold, is widely favoured due to its minimally invasive nature, quick recovery time, and cost-effectiveness [7]. However, these materials serve primarily as space fillers rather than tissue regenerators. Over time, resorption, migration, and foreign body reactions compromise their long-term effectiveness, often requiring repeat procedures [8]. For permanent correction, medialization thyroplasty is employed, wherein an implant is inserted to reposition the affected vocal fold [9]. Although effective, this method requires surgery, poses risks of infection, and does not address underlying tissue degeneration. Reinnervation techniques have also been explored, but they are often associated with variable clinical outcomes and long recovery periods [10]. The major limitation of current treatments is that they fail to restore the native vibratory properties of the vocal fold, which is essential for optimal voice production. Given these limitations, the field has shifted towards regenerative medicine, aiming to restore the biomechanical and structural integrity of the vocal folds.

Regenerative medicine is an interdisciplinary field that employs multidimensional approaches to repair and restore the native tissues adversely effected by congenital defects, ageing, diseases, or trauma. By using biomaterials, growth factors, and stem cells to promote the natural repair and remodelling of damaged tissues, this field offers a promising path toward vocal fold restoration [11]. Previous study observed that genipin-crosslinked gelatin hydrogel (GCGH) provides a stable and injectable platform for delivering regenerative molecules directly into the vocal fold tissue [12]. However, biomaterial alone is insufficient for complete tissue regeneration, prompting further investigation into biochemical factors that enhance the regenerative potential of hydrogel systems.

Among the various stem cell-based approaches, WJMSCs have gained significant attention due to their high proliferative capacity, immunomodulatory properties, and ease of extraction from umbilical cord tissue [13,14]. WJMSCs offer multipotency, allowing differentiation into fibroblasts, chondrocytes, and myocytes, which are crucial for vocal fold regeneration [15]. However, direct injection of stem cells has several drawbacks, including poor cell retention, rapid degradation, and difficulty in maintaining therapeutic concentrations at the injury site [16]. These challenges necessitate an advanced delivery system that sustains the therapeutic potential of WJMSCs while providing an optimal microenvironment for tissue repair. One such approach is the incorporation of WJMSCs within a hydrogel-based scaffold, which allows for localized, prolonged cellular activity and extracellular matrix (ECM) remodelling [17].

As of late, new developments in regenerative medicine have brought attention to the therapeutic potential of sEVs, which are membrane-bound, nanoscale vesicles released by cells that contain growth hormones, proteins, lipids, and ribonucleic acid (RNA) [18]. Moreover, sEVs serve as natural carriers of bioactive molecules, mediating cell-to-cell communication, and modulating tissue repair processes. Unlike stem cell transplantation, which poses risks of immune rejection and uncontrolled differentiation, extracellular vesicle (EV)-based therapies provide a safer, cell-free alternative, harnessing the regenerative capabilities of WJMSCs without the associated risks of tumorigenesis [19,20]. WJMSC-derived small EVs (WJMSC-sEVs) have demonstrated remarkable regenerative effects in various tissue engineering applications, including wound healing, neural repair, and cartilage regeneration [20,21].

Gelatin, an organic polymer primarily consisting of carbon, amino acids, and hydrogen, is derived from the controlled hydrolysis of collagen’s triple-helical structure via thermal or enzymatic processes [22,23]. This structure mimics the ECM through RGD motifs that support cell adhesion and biocompatibility, but its weak mechanical strength and rapid degradation limit long-term applications [22,24]. These drawbacks can be overcome using crosslinking strategies, with genipin offering an effective and biocompatible solution [25,26]. Extracted from *Gardenia jasminoides*, genipin reacts with amine groups in proteins to form stable covalent bonds, enhancing stability, strength, and resistance to degradation [27,28,29]. Notably, it is far less cytotoxic than conventional crosslinkers like glutaraldehyde, making it a safe candidate for tissue engineering, wound healing, and drug delivery [25,30]. Our previous studies characterized the physicochemical and biological properties of the GCGH with various formulation and found that 6% gelatin with 0.4% genipin offers a stable injectable platform for encapsulation of WJMSCs and bFGF, respectively, that support WJMSCs growth, sustain cell viability and migration, while maintaining minimal inflammatory response [12,26].

Thus, by co-encapsulating WJMSCs and sEVs within the selected GCGH formula, the current study aims to develop a multi-functional injectable scaffold capable of enhancing vocal fold fibroblast regeneration. This bioengineered hydrogel system has the potential to overcome the limitations of existing treatments, providing a sustained, localized regenerative effect while preserving the biomechanical properties of the vocal fold.

## 2. Materials and Methods

### 2.1. Tissue Processing

#### 2.1.1. WJMSC Isolation and Cryopreservation

Umbilical cord samples from the fetal segment were taken with consent from patients undergoing natural or elective surgery. Once collected, the sample was thoroughly disinfected with iodine and cleaned thoroughly using phosphate-buffered saline (PBS) (Gibco, San Diego, CA, USA). After the removal of arteries and veins, the tissue was minced and digested with 0.6% Collagenase Type I (Worthington Biochemical Corporation, Lakewood, NJ, USA) for 30 min to 1 h in incubator shaker at 37 °C. Then, the sample was washed with PBS and resuspend in Minimum Essential Medium Eagle—alpha modification (α-MEM) (Gibco, San Diego, CA, USA) supplemented with 10% fetal bovine serum (FBS) (Gibco, San Diego, CA, USA) and 1% antibiotic–antimycotic (AA) (Capricorn Scientific, Ebsdorfergrund, Germany). To create pooled WJMSCs stocks, nine batches of WJMSCs samples were grouped into three different pools, each combining three different batches of WJMSCs in equal ratios (1:1:1) at passage 2. These pools were expanded to passage 3, cryopreserved as master cell banks, and used for downstream characterization and experimentation at passages 4–6. Each batch of pooled WJMSCs was characterized according to a previously described protocol [31].

#### 2.1.2. VFFs Isolation

Vocal folds were collected from consented patients who underwent laryngectomee procedure. At 24 h after surgery, samples were immediately collected, processed, and cultured. Firstly, the vocal fold was washed with Dulbecco’s phosphate-buffered saline (DPBS) (Sigma-Aldrich, St. Louis, MO, USA) supplemented with 1% AA solution at least twice before submerged in iodine for 10 s. Then, it was washed again with DPBS until the solution became clear. Next, the vocal fold was cut into tiny pieces and added to 0.6% collagenase Type 1 solution. The sample was then digested by shaking it at 150 rpm and 37 °C for 20 min in an incubator shaker. After incubation, the sample was centrifuged at 5000 rpm for 5 min. The pellet was then washed with DPBS and centrifuged again. Lastly, the pellet was resuspended with Dulbecco’s Modified Eagle Medium/Nutrient Mixture F-12 (F-12 DMEM) (Gibco, San Diego, CA, USA) supplemented with 10% FBS and 1% AA. It was then transferred to a 6-well plate and cultured in incubator at 37 °C.

### 2.2. WJMSC-sEV Isolation and Characterization

WJMSCs were expanded and cultured in α-MEM containing 10% in-house produced human platelet lysate (HPL) and 1% AA [32], until it reached passage 4. At approximately 70% cells confluency, WJMSCs’ medium was changed to phenol red-free Dulbecco’s Modified Eagle’s Medium—Low Glucose (LG-DMEM) (Sigma-Aldrich, St. Louis, MO, USA) and incubated for 24 h at 37 °C. After that, 200 mL of cell culture medium was collected and centrifuged at 1300 rpm for 10 min. Then, it was filtered through 0.22 μm vacuum filter to remove large non WJSMC-EV particles. After that, WJSMC-EVs were isolated using Minimate™ EVO TFF System (Pall Laboratory, New York, NY, USA) with Minimate™ TFF Capsule with Omega Membrane-100 kDa (Pall Laboratory, New York, NY, USA) according to manufacturer’s manual. In brief, TFF capsules were initially flushed with milliQ water to remove free biomolecules. Then, excess milliQ was flushed out by adding sterile PBS solution. Following that, TFF system was recirculated with PBS to precondition the capsule and remove excess bubble. Then, culture medium was diafiltrated three times in PBS buffer. Finally, the solution was concentrated to approximately final volume of 7 mL and sterilized by 0.22 μm syringe filter to remove contaminants. Isolated WJMSC-sEVs were stored in freezer at −80 °C until further use.

#### 2.2.1. Concentration of WJMSCs Lysate, WJMSC-sEVs, and WJMSC-sEVs Lysate

Firstly, adherent WJMSCs were rinsed twice with cold PBS after conditioned media was collected. Then, WJMSCs were incubated with ice-cold radio immunoprecipitation assay (RIPA) lysis buffer (Thermo Fisher Scientific™, Boston, MA, USA) with 1% Halt™ protease and phosphatase inhibitor cocktail (Thermo Fisher Scientific™, Boston, MA, USA) for 5 min at 4 °C, followed by centrifugation at 14,000× *g* for 15 min at 4 °C. The supernatants were collected and stored at −80 °C for further use.

For WJMSC-sEV lysate preparation, EV were lysed with RIPA lysis buffer with 1% Halt™ protease and phosphatase inhibitor cocktail in a 1:1 ratio for 30 min at 4 °C and then centrifuged at 14,000× *g* for 15 min at 4 °C. The WJMSC-sEV lysate supernatants were transferred into 1.5 mL tube and kept at −80 °C until further use.

#### 2.2.2. BCA

Total protein concentration of WJMSC lysate, isolated WJMSC-sEVs, and WJMSC-sEV lysate was determined using Pierce^TM^ BCA Protein Assay Kit (ThermoFisher Scientific^TM^, Boston, MA, USA). The preparation of standards and working solutions were conducted according to the manufacturer’s protocol. Consequently, the solutions were transferred into a 96-well plate, covered and incubated at 37 °C for 30 min. Finally, the plate was read at 562 nm using a spectrophotometer (BioTek, PowerWave XS, Highland Park, IL, USA).

Total protein and total protein secreted per million cells were calculated using the following equations and tabulated in Section 3.1.2.

(1) Total protein concentration=Protein concentration×Final concentrated volume

(2) $$Total\;protein\;secreted\;permillion\;cells=\frac{Total\;protein}{Cell\;Number}$$

#### 2.2.3. Nanoparticle Tracking Analysis (NTA)

WJMSC-sEV size and number of particles were assessed using nanoparticle tracking analysis. An amount of 500 μL of WJMSC-sEV solution was introduced to NanoSight NS300 system (Malvern Instruments, Worcestershire, UK). The number of particles was captured during 60 s, and three captures were taken to obtain an average result.

Total particle count and number of particles secreted by each cell were calculated using the following equations and tabulated in Section 3.1.2.

(3) Total particles count=Particles Concentration×Final concentrated volume

(4) $$Number\;of\;particles\;secreted\;per\;cell=\frac{Total\;particles\;count}{Total\;Cell\;Number}.$$

#### 2.2.4. Western Blot

After obtaining total protein concentration of all samples, it was then calculated to desired concentration and then added to 5x sodium dodecyl sulphate (SDS) Loading Buffer (Elabscience, Wuhan, China). Then, the solution was boiled at 100 °C for 5 min before being stored on ice. After that, WJSMC-EVs proteins were electrophoresed through 10% sodium dodecyl sulphate polyacrylamide gel electrophoresis (SDS-PAGE) and transferred onto 0.45 μm pore sized Nitrocellulose Membranes (Thermo Fisher Scientific^TM^, Boston, MA, USA). Then, the membranes were blocked with tris-buffered saline with tween 20 (TBS-T) dissolved with 5% bovine serum albumin (BSA) for an hour. After the blocking step, the membranes were incubated individually with primary antibodies, which were rabbit monoclonal anti-CD63 (1:1000; Cell Signalling Technology, Danvers, MA, USA), rabbit monoclonal anti-TSG101 (1:1000; Cell Signalling Technology, Danvers, MA, USA), rabbit monoclonal anti-Grp94 (1:1000; Cell Signalling Technology, Danvers, MA, USA), and rabbit monoclonal anti-albumin (1:1000; Cell Signalling Technology, Danvers, MA, USA), respectively, at 4 °C overnight.

The membranes were then washed three times with TBS-T for 10 min each, before being incubated with anti-rabbit IgG secondary horseradish peroxidase (HRP)-linked antibody (1:5000; Cell Signalling Technology, Danvers, MA, USA) for an hour. Following that, the membranes were washed again with TBS-T thrice before being immersed in TBS solution. The membranes were then subjected to Pierce^TM^ Chemiluminescence (ECL) (Thermo Scientific^TM^, Boston, MA, USA) solution according to the manufacturer’s instruction. After that, all membranes were observed and recorded using Amersham Imager 600 gel documentation system (Cytiva, Marlborough, MA, USA).

#### 2.2.5. Transmission Electron Microscopy (TEM)

The morphology of WJSMC-EVs was also observed using a TEM. Prior to observation, negative staining was performed on WJMSC-sEVs. Firstly, a droplet of resuspended WJMSC-sEV solution was placed on carbon mesh (Agar Scientific, Stansted, UK) and left for 5 min. Then, it was gently dried with filter paper. Next, a droplet of 2% phosphotungstic acid (PTA) was dropped on it and left for 5 min. Finally, excess PTA was removed carefully with filter paper and placed in a dessicator for 72 h to ensure complete drying. Following the drying step, the carbon mesh was viewed inside the TEM (Leo Libra 120, Zeiss, Oberkochen, Germany) at an accelerating voltage of 1200 kV and total magnification of ×40,000.

### 2.3. Fabrication of Hydrogel

A combination of GCGHs was used as the basis of hydrogel for this research. Gelatin used originated from bovine bone, Type B (250 Bloom), produced by Nitta Gelatin India Limited, Cochin, India. As for genipin, the product was from FUJIFILM Wako Pure Chemical Inc., Osaka, Japan.

#### 2.3.1. Construction of GCGH

The formula for GCGH was selected and prepared based on the study by Ng et al. [26]. Briefly, 6% (*w*/*v*) gelatin was stirred in ultrapure water at 50 °C using a hotplate stirrer (Dragonlab, Raptor Supplies Pte Ltd., Singapore) until it was fully dissolved. Then, 0.4% (*w*/*v*) genipin was added into the solution and stirred for five minutes until it was fully homogenized. Lastly, the solution was transferred into the mould and left at room temperature until it was fully polymerized. This group was used as control in several experiments.

#### 2.3.2. Encapsulation of WJMSCs and sEVs in GCGH

WJMSCs at passages 4–6 were used in the fabrication with GCGH. The preparation of GCGH was as described in Section 2.3.1 but with additional steps. Prior to fabrication, WJMSCs were cultured, trypsinized, and resuspended in 100 μL of α-MEM medium. For GCGH-MSC, each 100 μL was calculated to a final cell density of 2 × 10^6^ cells/mL. In brief, 100 μL of WJMSCs was added into 900 μL of GCGH solution after it cooled down for about one minute. Then, the solution was resuspended and transferred to mould and kept at room temperature until it fully polymerized.

As for GCGH-sEVs, similar procedure was followed in Section 2.3.1. Briefly, after genipin was homogenized in the gelatin solution, the solution was taken off from the hotplate stirrer and left at room temperature for about one minute to cool down until about 37 °C. Then, a specific concentration of WJSMC-EVs (μg/mL) was added to the solution and mixed carefully before transferred into a mould.

### 2.4. Biocompatibility of WJMSC-sEVs and Selection Optimal Dosage

#### 2.4.1. Internalization of WJSMC-EVs in VFFs

This experiment was conducted to study the VFFs uptake of WJSMC-EVs. The nanoparticles were labelled by PKH26 red fluorescent dye (Sigma-Aldrich, St. Louis, MO, USA) according to manufacturer’s instruction. Briefly in 1.5 mL tube, 2 μL of PKH26 dye was added to 50 μg of WJMSC-sEV exosomes in 1 mL of diluent C. The tube was covered with aluminum foil and incubated for 10 min at room temperature. After incubation, the solution was transferred to Amicon^®^ Ultra Centrifugal Filter (100 kDa MWCO). Then, 2 mL of PBS was added to dilute the solution and centrifuged for 3 min at 4000× *g* and 4 °C. The dilution and centrifugation steps were repeated twice. For the final centrifugation, the solution was spun until it concentrated to a 50 μL volume. In addition, the negative control was prepared by combining sterile PBS to PKH26 dye.

Prior to the experiment, VFFs were seeded in a 48-well plate at cell density of 5000 cells/mL. Then, PKH26-labelled WJSMC-EVs and negative control were added to sub-confluent VFFs in a basal medium, separately. They were cultured for 12 h at 37 °C with 5% CO_2_ incubator. Then, the cells were washed twice with PBS and fixed with 4% paraformaldehyde (PFA) for 10 min at room temperature. After fixation, the cells were washed again thrice, each time for 5 min. Then, 6-diamidino-2-phenylindole (DAPI) (Cell Signalling Technology, Danvers, MA, USA) at concentration of 0.5 μg/mL was added to cells and incubated at room temperature for 10 min in dark surrounding. DAPI was then removed, and the cells were washed with PBS three times, each for 5 min. Lastly, the cells were observed, and images were captured at ×200 total magnification using Nikon AX/AX R Confocal Microscope System (Nikon, Tokyo, Japan).

#### 2.4.2. VFFs Proliferation Assay via MTT Assay

Prior to the experiment, VFFs (5000 cell/cm^2^) were seeded in a 48-well plate and cultured until they reached 70% confluency. Then, the cultured medium was changed to media with different concentrations of WJSMC-EVs (5, 10, 25, 50, 75, and 100 μg/mL, respectively). In addition, one positive control (complete medium without WJSMC-EVs) was added. After one day of incubation, the medium was removed, and cells were gently rinsed with DPBS. Next, 90 µL of pure basal medium with 10 µL of 5 mg/mL MTT (Sigma-Aldrich, St. Louis, MO, USA) solution was added to each well. Then the plate was incubated for 4 h at 37 °C. Next, 75 µL of the MTT solutions was discarded, and 50 µL of Dimethyl sulfoxide (DMSO) (Sigma-Aldrich, St. Louis, MO, USA) was added. The plate was shaken at 100 rpm in an incubator shaker at 37 °C for 10 min before the absorbance was read at 540 nm using a spectrophotometer. This assay was repeated for the experimental group incubated until day three and day seven. Percentage of cell viability was measured using the following calculation:

(5) $$Cell\;viability\;(\%)=\frac{At}{Ac}\times 100$$

At represents the absorbance of the treatment group and Ac represents the absorbance of the positive control group.

#### 2.4.3. Biocompatibility of VFFs with GCGH-sEVs via LIVE/DEAD^TM^ Assay

Subsequently, only three concentrations of WJMSC-sEVs were selected for further study. Four sets of GCGH were prepared, in which three sets were incorporated with different (50, 75, and 100 μg/mL) concentrations of WJSMC-EVs. The preparation of the GCGH and GCGH-sEVs was performed as reported previously by Ng et al. [26], with the addition of specific concentrations of sEV (50, 75, and 100 μg/mL).

Once GCGH and GCGH-sEVs were fully polymerized, VFFs were seeded on top of the hydrogel by placing at least 5 droplets of 10 μL cell suspension (5000 cells/μL). The hydrogels were carefully placed inside the incubator for 1 h to ensure VFF attachment. Then, complete medium was added around the hydrogel, avoiding contact with the area where the VFFs were seeded. Subsequently, the hydrogels were incubated for 48 h and 7 days, respectively, at 37 °C. Medium was changed every 2–3 days.

After incubation period, hydrogels were washed gently with 500 μL of DPBS, and the spent DPBS were collected to count the unattached cells. LIVE/DEAD^TM^ Viability/Cytotoxicity (Invitrogen, Waltham, MA, USA) solution was prepared fresh by combining calcein-AM and ethidium homodimer-1 at ratio of 1:4, in basal medium. The staining solution was then added to the hydrogels and incubated in total darkness for 30 min at 37 °C. Subsequently, the solution was taken out, and the hydrogels were soaked in α-MEM basal medium. The hydrogels were immediately viewed using Nikon Eclipse Ti fluorescence microscope (Nikon, Tokyo, Japan). Fluorescent images were captured at 10x magnification. All images were analyzed, and cells were counted using ImageJ software (Version1.53t, NIH, Bethesda, MD, USA). Notably, green-stained cells are viable cells, while red-stained cells are non-viable cells. VFF viability was calculated using the following formula:

(6) $$VFF\;Viability\;\left(\%\right)=\frac{Viable\;cells\;(green)}{Total\;cells\;(green+red)}\times 100\%$$

### 2.5. Physicochemical Properties of GCGH, GCGH-MSC, and GCGH-sEVs

Further into this study, three sets of GCGH-MSCs and GCGH-sEVs were introduced to observe their physicochemical properties, cellular interaction, and immunomodulatory effects as compared to GCGH alone.

Based on previous experiments (in Section 2.4), one concentration of sEVs was selected (75 μg/mL) for incorporation into the GCGH. The fabrication of all GCGH treatments was described in Section 2.3.

#### 2.5.1. Injectability Study

This study was conducted to observe the average time for of each hydrogel semi-solidification. An amount of 1 mL of hydrogel solution was prepared and transferred into the syringe with a 21 G needle gauge. Another 1 mL of hydrogel was also added to 1.5 mL Eppendorf tube as indicator. When the hydrogel did not drop in the inverted Eppendorf tube, the hydrogel in the syringe was extruded out with minimal force. Observations were noted every 5 min to see the viscosity, consistency, and flow properties of the hydrogel as it was injected out of the syringe.

#### 2.5.2. Biodegradation Study

Next, the hydrogels were weighed before being soaked in either 0.0006% (*w*/*v*) Collagenase Type 1 (Worthington Biochemical Corporation, Lakewood, NJ, USA) solution or Infusol^®^ HM Hartmann’s solution (Ain Medicare Sdn. Bhd., Kota Bahru, Malaysia). Then, the hydrogels were incubated at 37 °C and weighed every 24 h until they were fully degraded. The rate of biodegradation (mg/hour) was calculated using the following formula:

(7) $$Biodegaradation\;rate\;(mg/hour)=\frac{Wi\;(mg)-Wf\;(mg)}{Time\;(hour)}$$

### 2.6. Biocompatibility and Mechanistic Effect with GCGH Treatments

#### 2.6.1. Biocompatibility of VFFs via LIVE/DEAD^TM^ Assay

The cell viability of VFFs on the hydrogel surface were assessed by using LIVE/DEAD^TM^ Viability/Cytotoxicity kit. The methodology was the same as described in the previous section in 2.4.3. Briefly, VFFs were seeded on hydrogels and incubated for 2 and 7 days. After the incubation period, the hydrogels were washed and stained with LIVE/DEAD^TM^ dye for 30 min at 37 °C in the dark. After that, the dye was removed and replaced with complete medium before viewing using a Nikon Eclipse Ti fluorescence microscope. This experiment was repeated for the group of hydrogels incubated until day 7.

#### 2.6.2. Cytotoxicity of Hydrogel Leachate

To understand the cytotoxicity of hydrogel towards VFFs, hydrogel leachate was prepared and cultured together with VFFs. The preparation of leachate was based on ISO 10993-12 protocol (Biological evaluation of medical devices-Part 12, fourth edition, 10.3 Extraction conditions and methods), where 0.2 g of hydrogel immersed in 1 mL pure medium was incubated at 37 °C for 72 h. After collecting the leachate solution, it was filtered using 0.22 μm syringe filter to minimize impurities.

Next, VFFs were cultured in a 96-well plate at a density of 5000 cells/cm^2^. After 24 h of cultured, 100 μL medium with various concentration of leachate solution (20%, 60%, and 100%) was added into the wells. The plates were then incubated for 48 h at 37 °C. After incubation, VFFs viability was assessed using a MTT assay (Sigma-Aldrich, St. Louis, MO, USA). The leachate solution was removed, and the well was rinsed with DPBS. A total of 100 μL of basal medium with 10 μL of MTT solution was added to each well. The plate was incubated in the dark at 37 °C for 4 h. Then, 85 μL of MTT solution was removed, and 50 μL DMSO (Sigma-Aldrich, St. Louis, MO, USA) was added into each well. The plate was shaken at 100 rpm in a shaker incubator for 10 min at 37 °C. Lastly, it was read by spectrophotometer at optical density of 540 nm (BioTek, PowerWave XS, Highland Park, IL, USA). Cell viability was calculated using the following formula:

(8) $$Cell\;viability\;\left(\%\right)=\frac{At}{Ac}\times 100$$

At represents the absorbance of the treatment group and Ac represents the absorbance of the control group.

#### 2.6.3. Collagen Gel Contraction Assay

The protocol for this assay was based on Pincha et al. with some modification [33]. Firstly, VFFs were trypsinized and resuspended in complete F-12 DMEM supplemented with 10% FBS and 1% AA solution to a density of 1 × 10^5^ cells/mL. Then, the collagen gel was prepared by mixing 600 μL Rat-tail Collagen Type-I (Thermo Fisher Scientific, Waltham, MA, USA) solution with 400 μL cell suspension to obtain final concentration of 1 mg/mL. After that, the collagen solution was neutralized by adding 0.5 μL of 1M sodium hydroxide (NaOH). The collagen solution was kept on ice to slow down its polymerization. Then, the mixture was carefully pipetted up and down before being dispensed into a 24-well plate. The hydrogel was left to polymerized in incubator at 37 °C for 30 min. After hydrogels had polymerized, they were gently detached from the edges of the well with 10 μL pipette tips so that the hydrogels were able to contract freely. In each well, 200 μL of 20% leachate solution were added. An additional group of hydrogels with only complete F-12 DMEM media acted as positive control, while the negative control group consisted of collagen hydrogels without the incorporation of cells. Then, the plate was placed back into the incubator set at 37 °C with 5% CO_2_. The contraction of the hydrogels was monitored, and images were captured every 24 h. The diameters of the hydrogels were measured using ImageJ (Version1.53t, NIH, Bethesda, MD, USA) to assess the extent of contraction. The percentage of contractibility rates were calculated as follows:

(9) $$Contractibility\;rate\;\left(\%\right)=\frac{Ai-Af}{Ai}\times 100$$

Ai represents the initial area of collagen hydrogel, whereas Af represents the final area of collagen hydrogel.

#### 2.6.4. PBMCs Collection and Cell Culture

Whole blood was collected from healthy donors to isolate human PBMCs. Briefly, the blood was initially diluted with DPBS at a 1:1 ratio. Then, the diluted blood was carefully layered on Ficoll Paque Plus Density Gradient Media (GE Healthcare, Uppsala, Sweden) at a ratio of 2:1 (blood–ficoll paque). Next, the tubes were centrifuged at 1400× *g*, 25 ° C for 17 min. After that, the lymphocytes were collected from the interphase layer and washed three times with DPBS. After the washing step, the pellet was resuspended with Roswell Park Memorial Institute (RPMI)-1640 medium (Gibco, CA, USA) supplemented with 10% heat-inactivated FBS (Capricorn, Ebsdorfergrund, Germany) and 1% Penicillin–Streptomycin (Pen-Strep) (Gibco, CA, USA). The solution was then transferred to a T75 flask, incubated at 37 °C with 5% CO_2,_ and cultured for 24 h.

#### 2.6.5. PBMC Proliferation Using MTT Assay

After 24 h of resting, PBMCs were calculated and transferred into a 96-well plate with a cell density of 5 × 10^5^ cells/well. The treatment group contained complete RPMI (supplemented with 10% FBS and 1% Pen-Strep) medium with 20% leachate of GCGH, GCGH-MSCs, and GCGH-sEVs, respectively. The positive control group was supplemented with complete RPMI medium and 5 μg/mL of Phytohemagglutinin M-form (PHA-M)-form (Roche, Upper Bavaria, Germany) to stimulate lymphocyte proliferation, whereas the negative control group was cultured with RPMI medium containing 1% FBS.

After 24 h, cell viability was assessed with MTT assay (Sigma-Aldrich, St. Louis, MO, USA) to evaluate cell proliferation. Firstly, the medium was collected and transferred to 1.5 mL Eppendorf tube. The tubes were spun at 3000× *g* for 5 min. The pellets were washed gently with DPBS and spun again. Next, pellets were resuspended in 100 μL of MTT (5 mg/mL) solution before transferred to the wells. Then, the plate was incubated for 4 h at 37 °C in the dark. After incubation, 75 μL of MTT solutions was carefully removed, and 50 μL of DMSO was added to each well. Next, the plate was shaken at 100 rpm for 10 min. The absorbance was then measured using a spectrophotometer at 540 nm. The MTT assay wass also performed after 3 and 7 days of incubation.

### 2.7. Statistical Analysis

Each experiment was performed with three biological replicates, and each biological sample included three technical replicates. Each experimental group was tested in triplicate and replicated three times. All data were represented as mean ± standard deviation (SD). All data analysis were performed using by GraphPad Prism version 10.0 (GraphPad Software, Inc., San 51 Diego, CA, USA). One-way and two-way analysis of variance (ANOVA), as well as *t*-tests, were used to compare different values between the control group and the experimental group.

## 3. Results

### 3.1. Characterization of WJMSCs and WJMSC-sEVs

#### 3.1.1. WJMSCs Characterization

**Figure 1:**
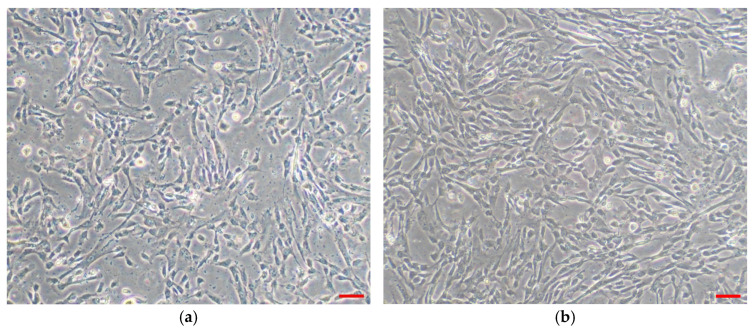
Morphology of WJSMC in tissue culture flask at ×100 magnification. (**a**) WJMSCs before pooling at passage 2. (**b**) Pooled WJMSCs at passage 4. Viewed under ×100 magnification; scale bar (red) represents 100 μm.

WJMSCs were isolated from Wharton’s Jelly of the umbilical cord. At passage 4, three biological samples were pooled into one. In accordance with International Society for Cellular Therapy (ISCT) guidelines, the pooled WJMSCs were characterized for their morphology, immunophenotype, and multilineage differentiation [34]. Figure 1 shown the morphological structure of WJMSC.

Pooled WJMSC phenotype was assessed using flow cytometry. Pooled WJMSCs expressed more than 95% (99.97 ± 0.02%) positive markers of CD73, CD90, and CD105 while expressing less than 2% (1.14 ± 1.07%) negative markers (HLA-DR, CD34, CD45, CD11b and CD19) (Table 1).

**Figure 2:**
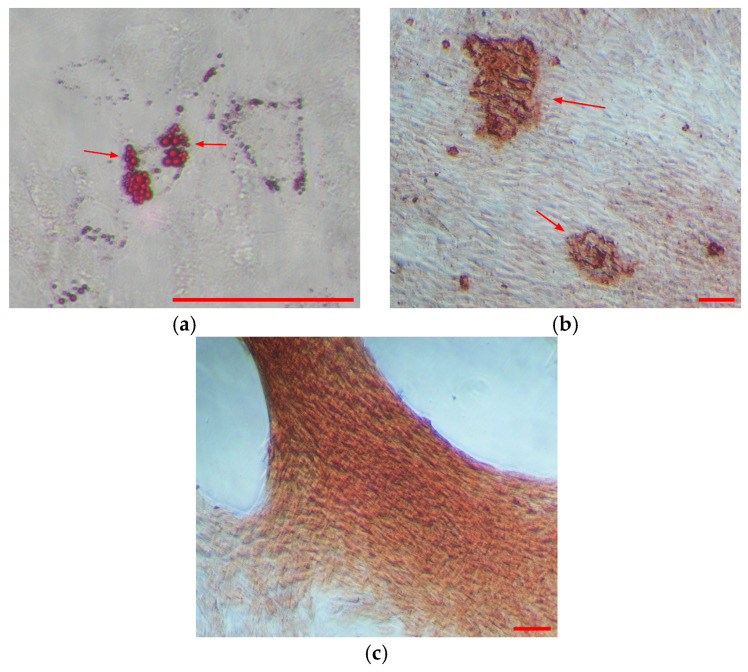
Multilineage differentiation of WJMSCs upon adipogenic, osteogenic, and chondrogenic induction. (**a**) Red arrows indicate oil red O-stained lipid droplet formation for adipogenic differentiation under ×200 magnification. (**b**) Red arrows show calcium deposition stained with alizarin red S for osteogenesis. (**c**) Chondrogenesis can be seen by formation of glycosaminoglycans stained with safranin O. Both (**b**,**c**) were viewed under ×100 magnification. Scale bar (red) represents 100 μm.

The differentiation of WJMSCs into adipocytes, osteoblasts, and chondroblasts was performed using the StemPro^TM^ Differentiation Kit. They were then stained with Oil Red O, Alizarin Red S, and Safranin O, respectively. In adipogenic differentiation, the formation of intracellular lipid droplets was observed, indicating the formation of adipocytes. As for osteoblast differentiation, calcium deposition was revealed after they were stained with alizarin red S. Lastly, chondroblasts were observed when safranin O stained the formation of glycosaminoglycans presented in the culture. Representative images from the staining show that WJMSCs have the capacity for trilineage differentiation (Figure 2).

#### 3.1.2. WJMSC-sEV Characterization

WJMSC-sEVs were isolated using the TFF system and characterized according to guidelines in MISEV 2018 [35]. Cell viability, cell count, and initial volume of condition media for each pooled WJMSCs were recorded (Table 2) to ensure consistency. The number of WJMSCs used to isolate sEVs was 62.33 × 10^6^ ± 13.27 × 10^6^, with cell viability of 97% ± 0.01. Consistent volume of condition media (200 mL) was also used to isolate sEVs.

After isolation of WJMSC-sEVs, the number of final concentrated volume of each pool of cells were tabulated in Table 3. Then, protein concentration was assessed by BCA Assay. Result shown that average concentration of isolated WJMSC-sEVs is 585.89 ± 298.94 µg/mL. Afterward, WJMSC-sEVs was characterized by NTA to measure the number of nanoparticles in each group and results were tabulated in Table 4.

**Figure 3:**
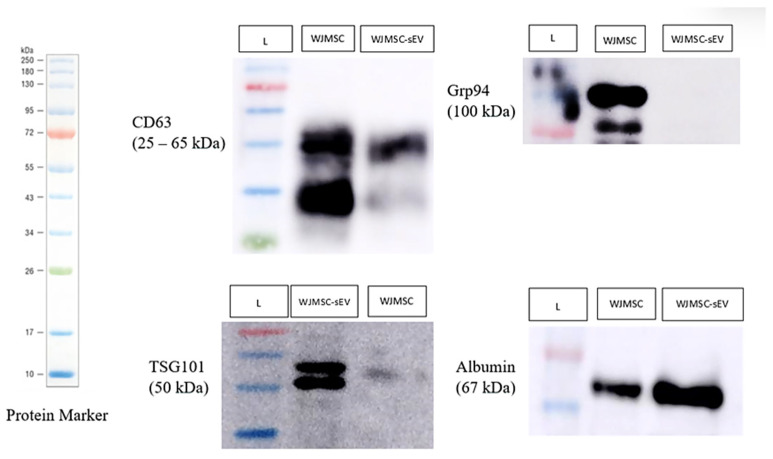
Immunoblotting analysis of WJMSC-sEV pool 2 for positive markers, CD63 and TSG101, negative marker, Grp94, and purity control marker, albumin. WJMSCs represent the cells, WJMSC-sEVs represent EVs, and L is the protein marker. Protein ladder (10~250 kDa) was used to determine and compare the sizes of each marker and samples.

In immunoblotting analysis, CD63 are usually observed on transmembrane of sEVs and TSG101 marked for cytosolic marker inside of sEVs. Grp94 was chosen as the negative marker for WJMSC-sEVs because the GRP94 mainly presents in endoplasmic reticulum. Besides positive and negative markers, albumin marked for the presence of non-EV co-isolated proteins. WJMSC-sEV suspension showed expression of CD63, TSG101, and albumin, while Grp94 was absent (Figure 3). The result showed that the sEV isolation is successful, but that WJMSC-sEV suspension contains albumin too.

**Figure 4:**
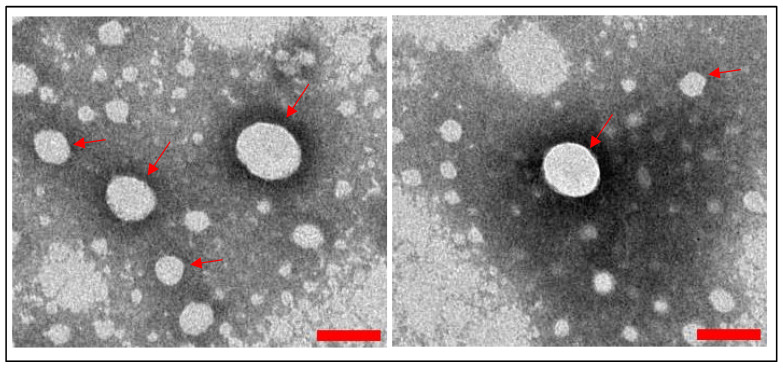
Morphology of WJMSC-sEVs viewed by TEM with ×40k magnification. Scale bar (red line) equals to 100 nm. Red arrows indicate WJMSC-sEVs.

Fully intact spherical shape of WJMSC-sEVs was observed at the nanoscale level (Figure 4).

### 3.2. Dose Selection of WJMSC-sEVs

Optimal concentration of sEVs was determined via three experiments (sEV uptake, cell proliferation, and cell viability) to evaluate the effect and compatibility of WJMSC-sEVs with VFFs.

#### 3.2.1. VFFs’ Ability to Uptake WJMSC-sEVs

**Figure 5:**
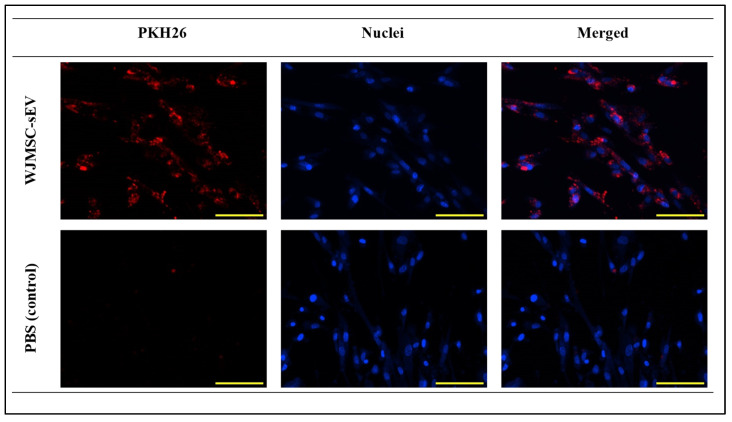
VFF uptake of PKH26 labelled WJMSC-sEVs and PBS (control). Red represents dyed PKH26 and blue represents nuclei stained with DAPI. Total magnification: ×100. Scale bar (yellow) indicates 100 μm.

Firstly, this experiment was performed to prove that WJMSC-sEVs are able to penetrate into VFF membrane. WJMSC-sEVs (50 μg/mL) were labelled with PKH26 dye, a lipophilic dye which binds to the bilipid layer of extracellular vesicles, prior to incubation with VFFs. For comparison, PBS was also stained with PKH26 dye as the negative control. PKH26 red spots were observed in VFFs incubated with WJMSC-sEVs, indicating that the WJMSC-sEVs were able to penetrate VFFs’ plasma membrane. At the same time, red spots were absent in the ones incubated with PBS (Figure 5). This is because there were no sEVs present in the PBS solution. This experiment demonstrated that WJMSC-sEVs were effectively internalized by VFFs.

#### 3.2.2. Proliferation of VFFs Cultured with Different Concentration of WJMSC-sEVs

**Figure 6:**
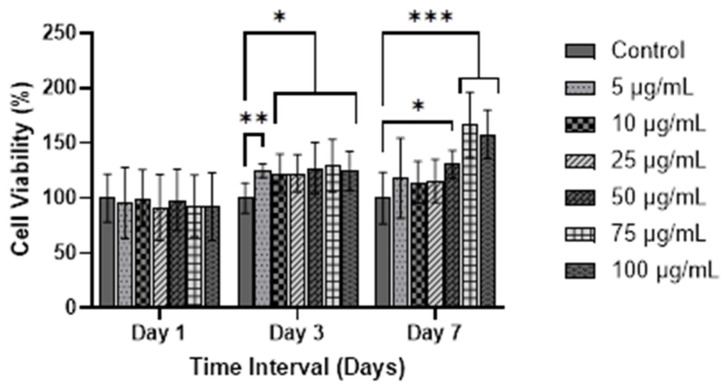
Proliferation of VFFs cultured in different concentrations of WJMSC-sEVs. Control was VFFs cultured in complete medium without WJMSC-sEVs (n = 9) * indicates *p* ≤ 0.05, ** indicates *p* ≤ 0.01, and *** indicates *p* ≤ 0.001 compared to control group. Statistics using two-way ANOVA.

VFFs proliferation was determined using the MTT assay to observe mitochondrial activity of cells on days 1, 3, and 7, as can be seen in Figure 6. The number of VFFs (5000 cells/cm^2^) cultured was kept consistent across all groups to ensure uniform growth. Compared to day 1, there were significant differences on day 3 for VFFs cultured with 5 μg/mL (124.9% ± 5.76), 10 μg/mL (122.1% ± 16.88), 25 μg/mL (122.59% ± 16.08), 50 μg/mL (127.34% ± 21.96), 75 μg/mL (129.37% ± 22.42), and 100 μg/mL WJMSC-sEVs (124.68% ± 16.82) compared to the control group (100% ± 12.9) (Figure 6). Eventually, on day 7, only 50 μg/mL (130.84% ± 11.83), 75 μg/mL (166.59% ± 28.11), and 100 μg/mL (158.16% ± 20.77) of WJMSC-sEVs exhibited higher cell viability when compared to the control group (100% ± 22.05). This demonstrates that the three concentrations of WJMSC-sEVs–50 μg/mL, 75 μg/mL, and 100 μg/mL—significantly promoted the proliferation of VFFs up to day 7.

#### 3.2.3. VFFs’ Compatibility with GCGH-sEVs

**Figure 7:**
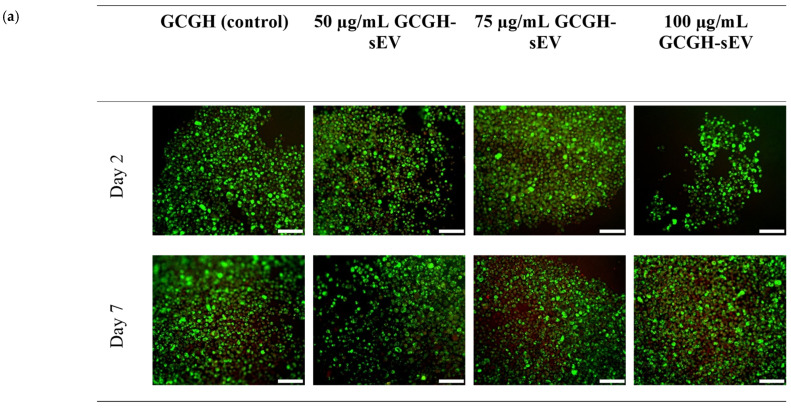

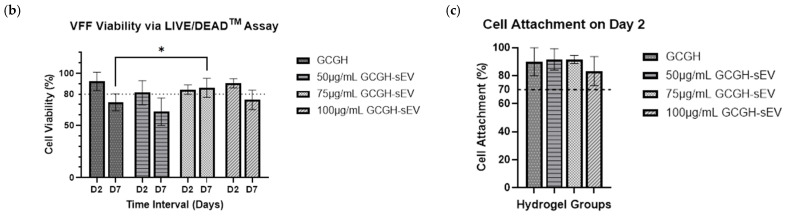
LIVE/DEAD^TM^ Viability/Cytotoxicity assay of VFFs. (**a**) Cell morphology, (**b**) cell viability, and (**c**) cell attachment of VFFs on GCGH-sEVs (n = 9) The cells were viewed under ×100 magnification, and each scale bar (white) represents 100 μm. * indicates *p* ≤ 0.05 compared to GCGH group. Statistical assay using two-way ANOVA (n = 3).

In this phase, three concentrations of WJMSC-sEVs from the previous experiment that showed significant difference by day 7 were chosen for encapsulation into GCGH for the LIVE/DEAD^TM^ assay. GCGH is the control group without the incorporation of WJMSC-sEVs. VFFs were seen to exhibit spherical morphology when seeded on the hydrogels (Figure 7). The percentage of cell viability at day 2 for GCGH (92.24% ± 8.28), 50 μg/mL GCGH-sEVs (81.41% ± 10.96), 75 μg/mL GCGH-sEVs (84.35% ± 4.25), and 100 μg/mL GCGH-sEVs (90.49% ± 4.23) were all above 80% range (Figure 7, b). As for the hydrogel groups incubated until day 7, only 75 μg/mL GCGH-sEVs (86.16% ± 8.55, *p* < 0.05) shows significant difference when compared to GCGH group and maintained cell viability above 80% compared to 50 μg/mL GCGH-sEVs (63.12% ± 12.39) and 100 μg/mL GCGH-sEVs (74.48% ± 8.72). All groups were found to have good initial cell attachment range between 83.33% ± 8.5 and 91.67% ± 2.36 before conducting the LIVE/DEAD^TM^ Assay.

### 3.3. Physicochemical Properties of GCGH, GCGH-MSCs, and GCGH-sEVs

In this part of the study, WJMSCs and WJMSC-sEVs were encapsulated in GCGH, respectively, and tested for injectability and in vitro biodegradability studies.

#### 3.3.1. Injectability Study

**Figure 8:**
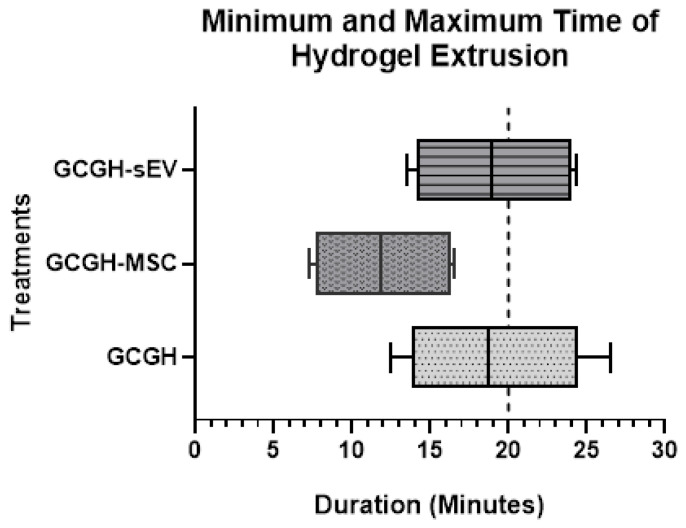
The boxplot dataset for hydrogel extrusion times for all three conditions (GCGH, GCGH-MSC, and GCGH-sEVs). The box indicates the optimum range for each hydrogel extrusion, and the line in the middle represents its median range. The lower whisker is the minimum time to dispense hydrogel, while the higher whisker shows the maximum time for hydrogel extrusion n = 9.

This study was performed to observe the duration of time for GCGH extrusion and estimate the best time for injection. The result shows that the median hydrogel extrusion for GCGH-MSCs (11.9 min) is lower than for GCGH (18.69 min) and GCGH-sEVs (18.9 min) (Figure 8). Each box also indicates its own lower whisker (GCGH; 12.49 min, GCGH-MSC; 7.26 min, GCGH-sEVs; 13.55 min) and upper whisker (GCGH; 26.54 min, GCGH-MSC; 16.59 min, GCGH-sEVs; 24.32 min), which are the minimum and maximum times, respectively, for each dataset. The interquartile ranges represent the gap between the minimum and maximum injection timepoint of each hydrogel group. GCGH (10.55 min) has a longer range compared to GCGH-MSCs (8.67 min) and GCGH-sEVs (9.96 min). At the same time, GCGH-MSCs is positioned at the left part of the chart compared to GCGH and GCGH-sEVs. But at the same time, the upper quartile of GCGH-MSCs overlapped with the lower quartile of GCGH and GCGH-sEVs. This means the hydrogels can be prepared at the same time and injected anytime between 12 and 16 min, as this is where they overlap. Overall, the boxplot shows that GCGH-MSCs (12.01 ± 3.72 min) has the shortest injection time compared to GCGH (19.1 ± 5.05 min) and GCGH-sEVs (19.09 ± 4.69 min), allowing for immediate injection.

**Figure 9:**
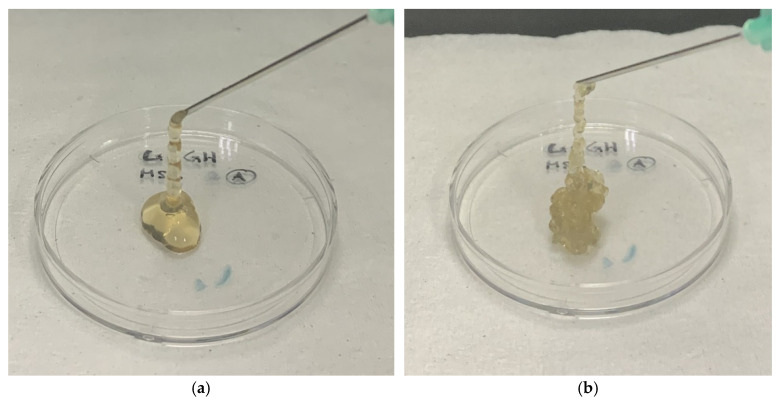
GCGH-MSCs extruded from syringe. (**a**) Hydrogel extruded at minimum timepoint. (**b**) Hydrogel extruded at maximum timepoint.

The physical differences in hydrogel extruded at minimum and maximum timepoints can be seen in Figure 9. At minimum timepoint, hydrogel extruded from the syringe appeared to form a consistent and uniform stream. Hydrogel extruded at maximum timepoint appeared to have uneven semi-solid strand with irregular consistency. Later timepoints will require more force for extrusion due to the GCGH being fully formed into a hydrogel. Thus, this study is essential for understanding and estimating the solidification time of GCGH, GCGH-MSC, and GCGH-sEVs when used as injectable materials.

#### 3.3.2. In Vitro Biodegradation

**Figure 10:**
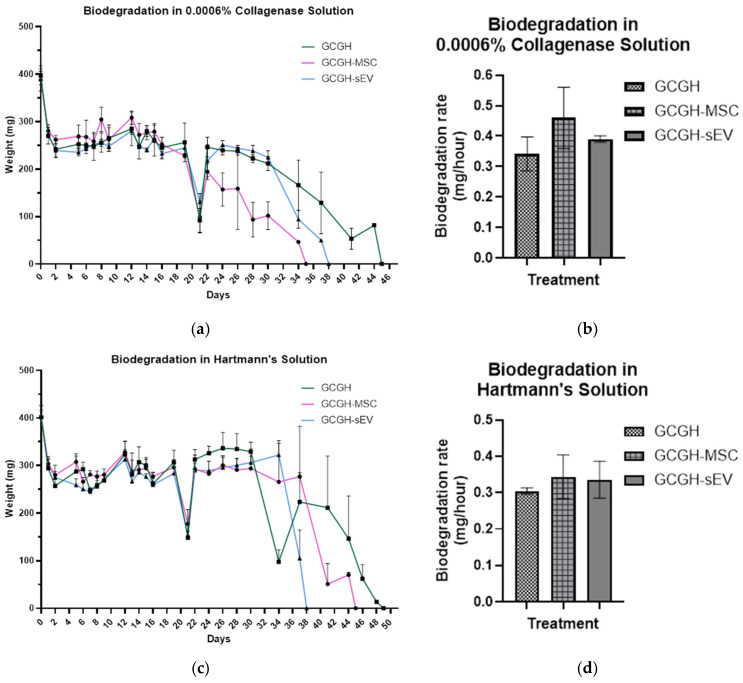
Biodegradation of GCGH, GCGH-MSC, and GCGH-sEVs. (**a**) In vitro degradation of treatments in 0.0006% collagenase solution over 44 days. (**b**) In vitro biodegradation rate of treatments in 0.0006% collagenase solution. (**c**) In vitro degradation of treatments in Hartmann’s solution over 48 days. (**d**) In vitro biodegradation rate of treatments in Hartmann’s solution (n = 9) Statistical analysis by one-way ANOVA.

In proteolytic degradation, GCGH-MSCs has the highest degradation rate (0.46 ± 0.08 mg/hour) and fully dissolves in 31.67 ± 3.29 days when compared to both GCGH (42 ± 1.41 days; 0.34 ± 0.05 mg/hour) and GCGH-sEVs (37 days; 0.39 ± 0.01 mg/hour) (Figure 10a,b).

As for hydrolytic degradation, GCGH, GCGH-MSC, and GCGH-sEVs were fully soaked in Hartmann’s solution until all hydrogels completely degraded. GCGH-sEVs has the fastest rate of degradation (0.34 ± 0.04 mg/hour) and all of them degraded at 37th day when compared to GCGH (46 ± 1.63 days; 0.31 ± 0.01 mg/hour) and GCGH-MSCs (43 ± 1.41 days; 0.34 ± 0.05 mg/hour) (Figure 10c,d). Although different groups degrade the fastest in each type of degradation, GCGH consistently degrades the slowest.

### 3.4. Biocompatibility of GCGH, GCGH-MSC, and GCGH-sEVs

#### 3.4.1. VFFs Compatibility by LIVE/DEAD Assay

**Figure 11:**
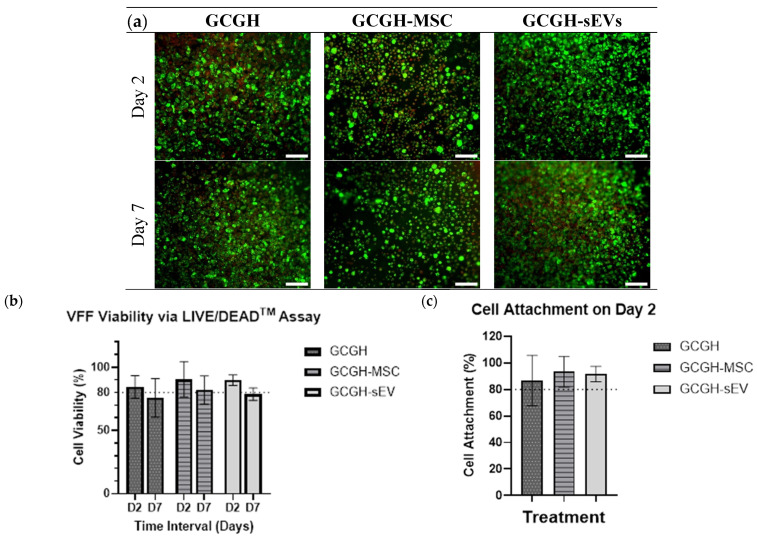
Cellular response via LIVE/DEAD^TM^ Viability/Cytotoxicity assay. (**a**) Cell morphology, (**b**) cell viability, and (**c**) cell attachment of VFFs on the hydrogels. The cells were viewed under ×100 magnification, and each scale bar (white) represents 100 μm. Statistical analysis using two-way ANOVA (n = 3).

All hydrogels show more than 80% cell viability on day two, especially GCGH-MSCs (90.48% ± 13.23) which show the highest percentage, followed by GCGH-sEVs (89.81% ± 3.97), and GCGH (84.46% ± 8.48). On day 7, GCGH (75.85% ± 14.45) and GCGH-sEVs (78.71% ± 4.68) were observed to decrease in cell viability below 80%, unlike GCGH-MSCs (82.04% ± 10.51). On both days, there is no significance difference between the groups (GCGH-MSCs and GCGH-sEVs) when compared to the GCGH group (Figure 11). It was also observed that all hydrogels have good VFF attachment with a range between 86.67% ± 15.45 and 93.33% ± 4.71.

#### 3.4.2. Cytotoxicity of Hydrogel Leachate to VFFs

**Figure 12:**
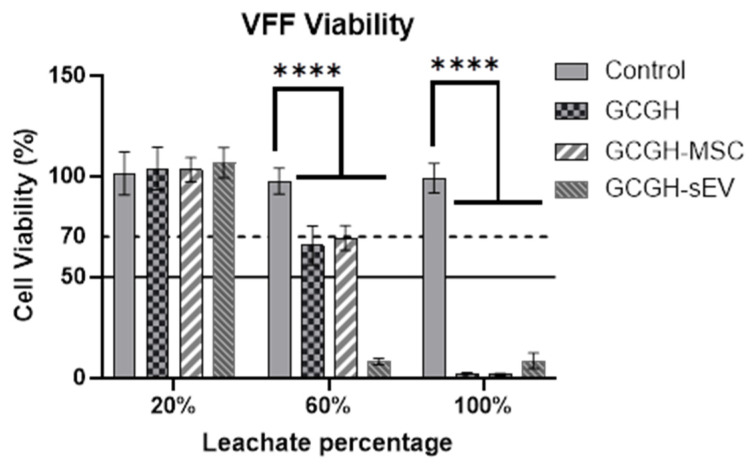
VFF viability cultured in different percentage of leachate solution. Control group is VFF cultured in complete medium (n = 9) Statistical analysis using two-way ANOVA. **** indicates *p* ≤ 0.0001 compared to control group.

The VFF groups cultured with 20% of the leachate solution of GCGH (103.83% ± 9.46), GCGH-MSCs (103.24% ± 5.38) and GCGH-sEVs (106.78% ± 6.76) have higher cell viability above the 70% threshold (Figure 12). Other concentrations of leachate solution exhibit cell viability below 70%.

#### 3.4.3. Collagen Gel Contraction Assay

**Figure 13:**
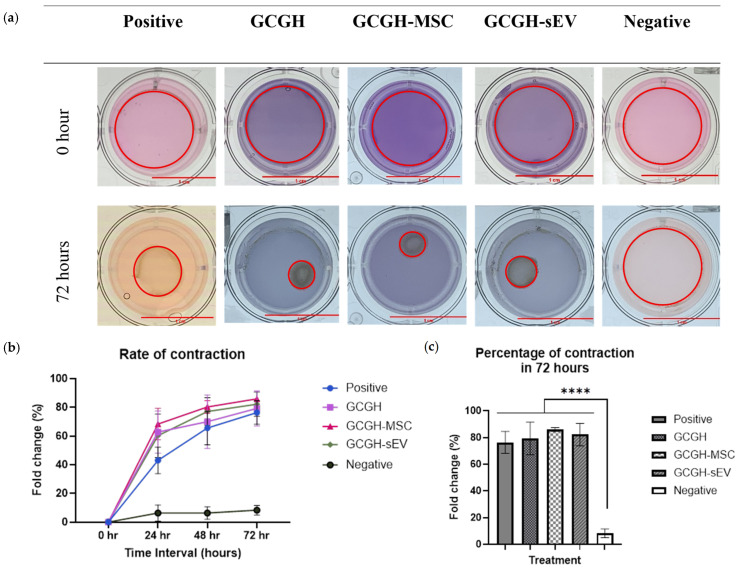
Collagen gel contraction assay of VFFs in mediums with GCGH, GCGH-MSC, and GCGH-sEV leachate. Positive control is VFF-incorporated collagen in complete medium. Negative control is collagen hydrogel without VFF incorporation. (**a**) Collagen hydrogel at 0 h and 72 h. (**b**) Rate of contraction in duration of 72 h. (**c**) Bar graph showing percentage of contraction at 72 h. Scale bar (red) = 1 cm (n = 9) Statistical analysis using two-way ANOVA. **** indicates *p* ≤ 0.0001 compared to negative control group.

A significant increment in percentage of fold change can be seen every 24 h (Figure 13). At 72 h, the results indicated that the overall percentage of fold change for GCGH (79.3% ± 10.54), GCGH-MSCs (85.98% ± 1.25), and GCGH-sEVs (82.22% ± 7.31) differed significantly from the negative control, as illustrated in Figure 13c.

#### 3.4.4. Immunomodulatory Response of PBMCs

**Figure 14:**
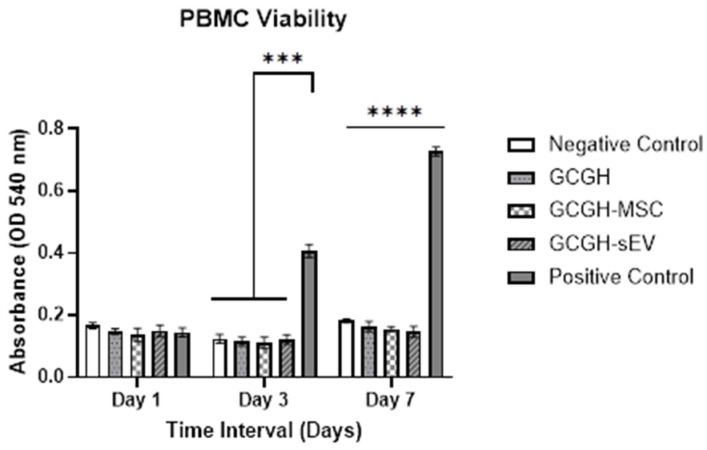
PBMC proliferation after culture with treatments and controls on day 1, 3, and 7. Positive control was induced with PHA-M, while negative control was cultured in RPMI medium with 1% FBS (n = 9) *** indicates *p* ≤ 0.001 and **** indicates *p* ≤ 0.0001 compared to positive control group; Statistics analysis using two-way ANOVA.

It can be seen in Figure 14 that not all treatments induced the proliferation of PBMC when compared to positive control (PBMC induced with 5 μg/mL of PHA-M). Significant differences in absorbance reading were observed on day 3 and day 7. On day 3, negative control (0.123 ± 0.011), GCGH (0.115 ± 0.011), GCGH-MSCs (0.11 ± 0.015), and GCGH-sEVs (0.121 ± 0.012) exhibited lower absorbance reading compared to the positive group (0.406 ± 0.017). The same reading was observed on day 7, when the positive control group (0.727 ± 0.011) had the highest absorbance compared to others. This proved that GCGH, GCGH-MSC, and GCGH-sEVs have non-immunomodulatory effect on PBMC.

## 4. Discussion

### 4.1. Characterization of WJMSCs and WJMSC-sEVs

In this study, three different batches of WJMSCs were pooled at passage 4 and characterized according to the ISCT guideline [34]. As expected, all the WJMSCs cultured from the umbilical cords of different donors were able to attach to tissue culture flasks even after they were pooled. In addition, flow cytometry results show that each pool expressed 95% (99.97 ± 0.02%) MSCs positive markers (CD73, CD 90, and CD105) and 2% (1.14 ± 1.07%) negative markers (HLA-DR, CD34, CD45, CD11b, and CD19) which is acceptable according to the guideline. In addition, the samples were able to differentiate into three different types of cells through adipogenesis, osteogenesis, and chondrogenesis [31]. These characterization results verified the identity of WJMSCs.

To ensure the successful isolation of sEVs from WJMSCs, characteristics of WJMSC-sEVs were assessed using NTA, Western blot, and TEM in accordance with Minimal Information for Studies of Extracellular Vesicles (MISEV) 2018 [35]. Firstly, NTA was used to obtain the concentration of nanoparticles including its particles sizes. According to the result, more than 90% of the particles fell within range (30–200 nm), which conformed to the WJMSC-sEV size range. Subsequently, to distinguish WJMSC-sEVs from other particles, protein markers were assessed using the Western blot analysis. The result shows that WJMSC-sEVs present transmembrane and cytosolic membrane markers specific to sEV (CD63 and TSG101, respectively) and the absence of Grp94, an endoplasmic reticulum marker. In addition, albumin, a serum contaminant, was also present in all isolated WJMSC-sEVs. The reason for this may be due to the TFF, a known powerful isolation tool, able to efficiently purify and concentrating sEVs without damaging the particles. However, TFF alone does not remove excess contaminants as efficiently as albumin [36]. According to a paper by Kawai-Harada et al., size exclusion chromatography (SEC) should be used to further purify EVs in order to eliminate undesired particles and debris [37].

Some studies suggest that the depletion of albumin cannot be performed due to its integration into sEV corona [38,39,40]. Sing et al. identified that albumin was one of 17 core proteins lost when the EV corona was disrupted [38]. In addition, Liam et al. found that when albumin was removed from the outer layer of EV, this resulted in a decrease in EV uptake in liver cells [40]. It shows that albumin may be an important part of sEVs in cell-to-cell attachments. While the effect of albumin on downstream experiments was not assessed in this study, it is concluded that the isolated WJMSC-sEVs are not pure.

In addition, TEM shows the morphology of WJMSC-sEV particles; when stained with PTA, they were heterogenous and spherical in shape, in line with the result observed by Forteza-Genestra et al. [41]. MISEV 2024 recommended staining with uranyl acetate or methylcellulose-based compounds to show the bilipid layer of the EVs [42]. This is because PTA shows low contrast compared to uranyl acetate and causes risk of artefact formation due to uneven drying of the samples [43]. The result from each characterization prove that the isolation of WJMSC-sEVs was successful.

This study did not dive into stability study for isolated WJSMC-sEVs, but after isolation, the concentrated solutions were directly kept at −80 °C in small batches to avoid multiple freeze–thaw attempts. At present, research on the long-term storage of WJMSC-sEVs is limited; however, a researcher observed that storing UCMSC-sEVs at −80 °C for up to one month efficiently retained sEV particle sizes, protein markers, and biological functionality when tested in sarcoma cell proliferation [44]. Similarly, another researcher found that by storing UCMSC-sEVs by first lyophilizing or encapsulating them in hyaluronic acid, they were able to preserved its structure and bioactivity after two months of storage at −80 °C [45]. While long-term stability of WJMSC-sEVs remains questionable in this study, further experiments incorporating WJMSC-sEVs into GCGHs provide significant results when compared to the GCGH-only group.

### 4.2. Selection of WJMSC-sEV Concentration

EVs are known to be internalized by target cells and subsequently deliver signals that promote cell proliferation and immunosuppressive effects [46]. It was observed that VFFs were able to uptake WJMSC-sEVs when it was added exogenously to medium and cultured for 12 h. The internalization of WJMSC-sEVs was visualized through confocal microscopy with red-fluorescence, indicating the presence of WJMSC-sEVs, while blue denoted the nuclei of VFFs. Since sEVs are small nanoparticles and observation using standard confocal microscopy is difficult, staining sEVs with PKH26 dye has been helpful in tracking their presence [47]. PKH26 is a lipophilic dye that has a long hydrophobic tail that easily bounds to the lipid bilayer of sEVs, showing as a red, bright fluorescent colour [48]. Even though tracking WJMSC-sEVs is possible, explanation on its internalization pathway remains a mystery. Several theories have been proposed, including the endocytic pathway, clathrin-independent pathway, micropinocytosis, phagocytosis, and lipid raft-mediated internalization [49]. In addition to this, it also suggested the involvement of CD9 and CD63 marker on the uptake of sEVs into target cells [50]. But, another group argues that these markers are not involved in this pathway by providing experimental result using two independent assays on different cell lines [51]. Thus, the mechanism of action of sEV internalization remains debatable, and this matter could be further explored in the future in order to gain more understanding.

Currently, the number of doses in current clinical trials varies from one disease to another [52]. Furthermore, studies combining scaffolds with sEVs are limited, with different concentration of sEVs incorporated in different types of biomaterials. For example, a study showed that incorporating 20 μg/mL of umbilical cord-derived mesenchymal stem cells (UCMSC)-EV in gelatin methacrylate (GelMA) hydrogel promotes cell regeneration and angiogenesis in diabetic wound healing [53]. According to a different study by Chen et al. [54], chondrocytes cultivated in 15 μg/mL of UCMSCs stimulate increased cell proliferation 1.2 times more than their control group across four doses (1 μg/mL, 5 μg/mL, 10 μg/mL, and 15 μg/mL).

It is hypothesized that higher concentration of WJMSC-sEVs enhance cell proliferation. So, this study proposes six different concentrations of sEVs and observes their proliferative and cytotoxicity effect on VFFs. Initially, WJMSC-sEVs at various concentrations (5, 10, 25, 50, 75, and 100 μg/mL) significantly enhanced the proliferation of VFFs at day 3 compared to the control group. Subsequently by day 7, only the 50, 75, and 100 μg/mL concentrations maintained significantly higher proliferation rates.

Then, to further evaluate and narrow down the selection of sEV concentration, the LIVE/DEAD^TM^ assay was performed using three different concentrations of sEVs (50, 75, and 100 μg/mL) incorporated into GCGH. From the result, 75 μg/mL maintain better VFF viability until day 7 compared to WJMSC-sEVs 50 μg/mL and 100 μg/mL. It is noticeable that 75 μg/mL had a better effect on VFF proliferation compared to other concentrations, even though the initial hypothesis was that the higher concentration of sEV directly proportional to better cell proliferation. The efficiency of sEV concentration is still being explored, and more research should be performed to clearly understand it, especially in clinical trials.

### 4.3. Incorporation of Biological Components in GCGH on VFFs Compatibility

As a natural crosslinker, genipin polymerizes gelatin by attacking its C3 carbon ring with primary amine groups from gelatin. This finally opens the ring, allowing ester replacement with another amine group and forming covalent bridges between two gelatin chains [55]. A persistent three-dimensional hydrogel network is produced by this repeating reaction, which frequently has a distinctive blue coloration from the oxidative reaction during genipin self-polymerization [55,56].

This study used Ng et al.’s group formulation in producing GCGH as biomaterial base for the incorporation of WJMSCs and WJMSC-sEVs [12,26]. According to their research, hydrogel containing 6% gelatin and 0.4% genipin had the best conditions as an injectable hydrogel taking less than 20 min to polymerize, having a low swelling ratio, degrading slowly in vitro, and having an elastic modulus between 2 and 10 kPa [26]. Additionally, it was found that pore sizes between 100 and 400 μm sustained WJMSC motility, attachment, and viability for over 7 days with low inflammatory reaction [12].

To ascertain the best timing for injection in a clinical setting, the injectability of GCGH groups was assessed. The gelation process is time-sensitive because slow crosslinking will cause the biomaterial to disperse in place, but rapid crosslinking will probably block the needle and prevent it from extruding [57]. Furthermore, to be performed in a clinical context, the GCGH solution-to-gel transition should take less than 20 min. From the result, it is seen that GCGH-MSCs had the shortest extrusion time (12.01 ± 3.72 min), while GCGH and GCGH-sEVs had an extraction time of about 19 min, suggesting differences in viscosity and gelation kinetics.

Biodegradation analysis of GCGH is important to determine the duration of hydrogel degradation while concurrently estimating the delivery of WJMSCs and sEVs for vocal fold regeneration. In this study, collagenase enzyme was used in proteolytic degradation test to imitate in vivo ECM resulting in gradual disposition of biomaterials into solution [25]. It is revealed that GCGH-MSCs degraded the fastest under proteolytic conditions (31.67 ± 3.29 days). This process occurred due to enzymatic reaction of collagenase that cleaved intermolecular bonds between gelatin and genipin [58]. In addition, hydrolytic degradation was assessed to cause the breakdown of large molecules, for example, gelatin, into smaller molecules when reacting to water. This is important in medical implants, such as bioscaffold and injectable polymers, designed to gradually breakdown within the body after it fulfilled its role [59]. This experiment uses Hartmann’s solution usually administered to patients to replace lost fluids and electrolytes in the body. By immersing GCGH groups in Hartmann’s solution, it was observed that GCGH-sEVs degraded the fastest (0.34 ± 0.04 mg/hour) in hydrolytic conditions. The reason behind this is the presence of PBS in which EVs were stored. This increases the number of water molecules in GCGH-sEVs, subsequently speeding up the process of degradation compared to others. This suggests that WJMSCs and sEV incorporation may influence hydrogel stability and degradation patterns, which can be fine-tuned for controlled release applications.

### 4.4. Biocompatibility of VFFs with GCGH Groups

Important factors in choosing suitable scaffold in tissue regeneration are biocompatibility and low cytotoxicity of by-products during degradation. This study evaluated biocompatibility among GCGH groups with VFFs through cytotoxicity and viability assays. The LIVE/DEAD assay demonstrated that all hydrogels supported initial cell attachment (~86.67% to 93.33%) and maintained viability above 80% until day 2. However, by day 7, only GCGH-MSCs maintained viability above this threshold, suggesting superior cell-supportive properties. This might be due to the concentration of WJMSCs (2 × 10^6^ cells/mL) incorporated in GCGH as compared to concentration of WJMSC-sEVs (75 μg/mL). WJMSCs encapsulated in hydrogels enhance cell proliferation by providing release of secretomes including sEVs [60,61,62]. Cytotoxicity assays using hydrogel leachate also indicated that only 20% leachate concentration was non-toxic to VFFs. A few reasons for this can be suggested, including VFFs cellular characterization and proteomics. A study by Karbiener et al. [63] compared VFFs with oral mucosa fibroblasts (OMFs) and found that VFFs proliferated more slowly and were larger in size. Furthermore, compared to OMFs, which have elevated proteins specifically connected to cellular proliferation, nuclear events, and defence against oxidative stress, VFFs contain more factors focusing on ECM repair, according to a proteomics study [63]. In the study, it was observed that VFFs cultured on GCGH exhibit significant reduction in cell proliferation compared to other hydrogels. Therefore, the incorporation of WJMSCs and WJMSC-sEVs remains necessary since they can maintain VFFs viability. But the results reinforce the need for optimized hydrogel formulations to prevent adverse effects from degradation byproducts.

To better visualize the mechanical effect of VFFs cultured with GCGHs, collagen contraction (Col-Gel) lattice assay was performed. VFFs were incorporated in Col-Gel to mimics vocal fold function in terms of contractibility [64]. This process mimics how cells interact with ECM in vivo, when mechanical force is applied to remodel environment. Furthermore, floating matrix model of Col-Gel assay was adapted, which is known to facilitates fibroblast migration in the initial stage of wound contraction [65]. It was observed that GCGH-MSCs induce better VFF Col-Gel contraction compared to other groups. Similarly, it was reported that bone marrow-derived MSCs conditioned media enhanced Col-Gel contraction which had a favourable effect on ECM regulation [66]. In comparison, GCGH-only leachate exhibited lowest contractility, almost the same as the positive control. This further supported the functional impact of WJMSCs and WJMSC-sEVs in demonstrating significant VFF-mediated contraction, a critical feature in extracellular matrix remodelling. The observed contraction in WJMSC and sEVs treated groups suggests a role in promoting fibroblast-mediated tissue repair.

Finally, the immunomodulatory response of PBMCs was assessed by co-culturing them with GCGH groups and compared them against a positive control condition which is PHA-M. It was shown that when PBMCs were cultured with GCGH groups, they did not exhibit significant proliferation, but when cultured with PHA-M, the proliferation of PBMCs significantly increased on day 3 and day 7. Stimulating PBMC proliferation requires one of these wo signals, mitogenic stimulation or cytokines. *Phaseleolus vulgaris*, or red kidney beans, contain the plant lectin protein PHA-M, which is the mucoprotein form of PHA. It is a recognized mitogen that promotes PBMC development by forming crosslinks with glycoproteins on T-cell surfaces [67]. In contrast, the GCGH groups do not provide similar receptor engagement or co-stimulatory signals to induce any immune cells in PBMC culture. The incorporation of GCGH with WJMSCs did not have an effect on the proliferation of PBMCs. This is due to the immunomodulatory activities of WJMSCs in the GCGH, which decrease PBMC activation by secreting anti-inflammatory cytokines such IL-10 and TGF-β [68]. As for sEVs, depending on their cargo, they contains a mix of signalling molecules including micro ribonucleic acid (miRNA) and proteins. Previous study found that sEVs derived from UCMSCs has the same immunosuppressive as it parent cells, which inhibit the proliferation of PBMCs [69]. The lack of PBMC response to GCGH-groups supports its potential in translation into a clinical setting.

## 5. Conclusions and Future Prospect

This study demonstrated the therapeutic promise of a novel biomaterial combining GCGH with small extracellular vesicles derived from WJMSC-sEVs for VFF regeneration. Prior characterisations of WJMSC-sEVs are important to highlight their particle size range, specific protein markers, and presence of bilipid layer morphology to differentiate WJMSC-sEVs from other non-EV particles or debris. The integration of WJMSC-sEVs with GCGH significantly enhanced VFF proliferation, preserved cell viability, and positively influenced hydrogel degradation rates. Additionally, the biomaterial showcased ideal properties such as biocompatibility, injectability, and biodegradability—making it an excellent scaffold for regenerative applications. Notably, both WJMSCs and WJMSC-sEVs modulated ECM remodelling, with WJMSCs showing the most robust effect despite contrary expectations.

Mechanistic insights from collagen gel contraction assays confirmed improved matrix remodelling in groups treated with GCGH supplemented with WJMSC-related elements. These findings support previous literature suggesting that sEV carry miRNAs and growth factors crucial for tissue repair. Immunomodulatory assessments using PBMC co-culture assays further validated the safety profile of the hydrogel composites, as none of the tested groups induced significant immune activation. Additionally, GCGH’s sustained degradation profile supports long-term therapeutic use, allowing for prolonged bioactive molecule release. Overall, the engineered hydrogel created a favourable environment for fibroblast adhesion and function, highlighting its potential in vocal fold regeneration and broader soft tissue engineering.

However, several limitations must be acknowledged. The study was conducted entirely in vitro, limiting its immediate translational relevance to clinical settings. The TFF method used for sEV isolation did not eliminate protein contaminants such as albumin, which may influence cell responses. Moreover, the short duration of the study restricts understanding of long-term cellular behaviour, matrix remodelling, and scaffold stability. Variability in sEV content due to donor heterogeneity and purification inconsistencies further challenges the reproducibility of results. These aspects underscore the need for more refined isolation techniques and standardized protocols for sEV production and storage.

To advance toward clinical translation, future research should include in vivo studies to evaluate the long-term safety and regenerative efficacy of GCGH-sEVs in animal models. Further exploration into the molecular pathways influenced by WJMSC-sEVs, especially the role of specific miRNAs and proteins in ECM remodelling, could enhance our understanding of its mechanisms of action. Standardization efforts in producing sEVs and immune response studies are critical to ensure safety and consistency across applications. In conclusion, while challenges remain, this study lays essential groundwork for the development of injectable, stem cell-derived biomaterials, with the potential to transform treatment approaches for vocal fold damage and other soft tissue injuries.

## Figures and Tables

**Table 1:** Immunophenotype of WJSMCs by flow cytometry. Percentage of events for each pooled WJMSCs.

Markers	Events (%)		
	Pool 1	Pool 2	Pool 3
CD90+	100	99.95	99.95
CD105+	99.67	96.9	96.19
CD73+	97.45	99.59	99.55
Negative	2.63	0.6	0.19

**Table 2:** Quantitative assessment of WJMSCs and protein concentration of WJMSCs lysate.

Pool	1	2	3	Mean ± SD
Total cell number	44 × 10^6^	75 × 10^6^	68 × 10^6^	62.3 × 10^6^ ± 13.23 × 10^6^
Cell viability (%)	95%	98.2%	96.3%	97% ± 0.013
Protein concentration of cell lysate (µg/mL)	1779.6	2025.78	1745.07	1850.14 ± 124.99
Initial total CM volume (mL)	200	200	200	n/a

**Table 3:** Final concentrated volume of WJMSC-sEVs, protein concentration of WJMSC-sEVs and WJMSC-sEV lysate, and total protein of WJMSC-sEVs.

Pool	1	2	3	Mean ± SD
Final concentrated volume (mL)	9	7.5	8	8.17 ± 0.62
Protein concentration of sEVs (µg/mL)	208.97	940.159	608.545	585.89 ± 298.94
Protein concentration of sEV lysate (μg/mL)	425.4	1044.31	673.64	714.45 ± 254.31
Total protein of sEV lysate (mg)	3.84	7.83	5.39	5.68 ± 1.64
Total protein per 10^6^ cells (μg/10^6^ cells)	87.01	104.43	79.25	90.23 ± 10.53

**Table 4:** Nanoparticle tracking analysis of WJMSC-sEVs.

Pool	1	2	3	Mean ± SD
Concentration (particles/mL)	3.9 × 10^10^ ± 6.01 × 10^9^	5.55 × 10^10^ ± 5.81 × 10^9^	4.95 × 10^10^ ± 1.09 × 10^10^	4.8 × 10^10^ ± 0.68 × 10^10^
Total particle count	3.51 × 10^11^	4.16 × 10^11^	3.96 × 10^11^	38.76 × 10^10^ ± 2.71 × 10^10^
Mode size (nm)	65.1 ± 2.7	63.4 ± 4.4	72.7 ± 2.7	67.07 ± 4.04
Mean size (nm)	89.2 ± 2.0	84.0 ± 2.5	91.2 ± 1.1	88.13 ± 3.03
Number of particles secreted per cell	7.98 × 10^3^	5.55 × 10^3^	5.82 × 10^3^	6.45 × 10^3^ ± 1.09 × 10^3^

## Data Availability

All original contributions from this study are provided in the article. Please contact the corresponding author for further inquiry.

## Abbreviations

AA	Antibiotic-Antimycotic
ANOVA	Two-Way Analysis of Variance
BSA	Bovine Serum Albumin
BCA	Bicinchoninic Acid Assay
CaHA	Calcium Hydroxyapatite
Col-Gel	Collagen Contraction
DAPI	6-diamidino-2-phenylindole
DMEM F-12	Dulbecco’s Modified Eagle Medium/Nutrient Mixture F-12
DMSO	Dimethyl sulfoxide
DPBS	Dulbecco’s phosphate-buffered saline
ECM	Extracellular Matrix
ECL	Chemiluminescence
EV	Extracellular Vesicles
FBS	Fetal Bovine Serum
F-12 DMEM	Dulbecco’s Modified Eagle Medium/Nutrient Mixture F-12
FRGS	Fundamental Research Grant Scheme
GCGH	Genipin Crosslinked Gelatin Hydrogel
GCGH-MSC	Genipin Crosslinked Gelatin Hydrogel-Mesenchymal Stem Cells
GCGH-sEVs	Genipin Crosslinked Gelatin Hydrogel-Small Extracellular Vesicles
GelMA	Gelatin Methacrylate
HA	Hyaluronic Acid
HPL	Human Platelet Lysate
ISCT	International Society for Cellular Therapy
LG-DMEM	Dulbecco’s Modified Eagle’s Medium-Low Glucose
MISEV	Minimal Information for Studies of Extracellular Vesicles
miRNA	Micro Ribonucleic Acid
MoHE	Minister of Higher Education
MSCs	Mesenchymal Stem Cells
MTT	3-(4,5-dimethylthiazol-2-yl)-2,5-diphenyltetrazolium bromide
NaOH	Sodium hydroxide
NTA	Nanoparticle Tracking Analysis
OMF	Oral Mucosa Fibroblasts
PBMCs	Peripheral Blood Mononuclear Cells
PBS	Phosphate-Buffered Saline
Pen-Strep	Pennicilin-Streptomycin
PFA	Paraformaldehyde
PHA-M	Phytohemagglutinin M-form
PTA	Phosphotungstic Acid
RIPA	Radio Immunoprecipitation Assay
RNA	Ribonucleic Acid
RPMI	Roswell Park Memorial Institute-1640 medium
SDS	Sodium dodecyl Sulphate
SDS-PAGE	Sodium Dodecyl Sulphate Polyacrylamide Gel Electrophoresis
SEC	Size Exclusion Chromatography
sEV	Small Extracellular Vesicles
TBS-T	Tris-Buffered Saline with Tween 20
TEM	Transmission Electron Microscopy
TFF	Tangential Flow Filtration
UCMSC	Umbilical Cord-Derived Mesenchymal Stem Cells
UKM	Universiti Kebangsaan Malaysia
VFFs	Vocal Fold Fibroblasts
WJMSCs	Wharton’s Jelly mesenchymal stem cells
WJMSC-sEVs	WJMSC-Derived Small EV
α-MEM	Minimum Essential Medium Eagle-Alpha Modification
